# Navigating the Diagnostic Challenges of Colloid Milium: A Case Report

**DOI:** 10.7759/cureus.116798

**Published:** 2026-09-23

**Authors:** Malika Oblokulova, Malika Solmetova, Mark Chumack, Yana Turkowski

**Affiliations:** 1 Department of Dermatology, Central Asian University, Tashkent, UZB; 2 Department of Dermatovenereology and Cosmetology, Tashkent State Medical University, Tashkent, UZB; 3 Department of Biomedical Engineering, University of Massachusetts Amherst, Amherst, USA; 4 Department of Medicine, VA Boston Healthcare System, Boston, USA

**Keywords:** chloasma, colloid degeneration, colloid milium, cutaneous hyalinosis, dermal papillae deposits, eosinophilic substance, histological diagnosis, misdiagnosis, rare dermatological condition, sun exposure

## Abstract

Colloid milium (CM), also known as cutaneous hyalinosis or colloid degeneration of the skin, is an underreported dermatological condition characterized by the accumulation of eosinophilic, homogeneous deposits in the dermal papillae. This case report presents an instance of CM in a 48-year-old female patient who exhibited yellow-brown papules confluent into plaques on the face, neck, and dorsal hands. This case is unique because CM presented with a clinical appearance resembling chloasma, resulting in a diagnostic challenge. Initially misdiagnosed and ineffectively treated as chloasma, the condition remained unrecognized for several years, further complicating her cosmetic concerns. The atypical presentation highlights the difficulty of relying solely on clinical morphology and underscores the importance of maintaining a broad differential diagnosis. Histopathological examination was essential for establishing the definitive diagnosis of CM, marking a crucial turning point in her treatment journey. Despite the general understanding that prolonged sun exposure plays a significant role in the pathogenesis of CM, the precise mechanisms remain unclear, making this case a valuable addition to the ongoing exploration of its etiology. This case serves as a reminder of the need to improve clinical recognition of atypical presentations of CM and emphasizes the important role of histopathology in confirming the diagnosis.

## Introduction

Colloid milium (CM) is an unusual and distinctive cutaneous deposition disorder, first described by Dr. Ernst Wagner in 1866. CM, also termed “colloid degeneration” and “elastosis colloidalis conglomerata,” includes a group of conditions characterized by dermal deposits of hyaline-like, homogeneous eosinophilic material [1-3]. There are limited epidemiological data on the prevalence of this condition [4].

The colloid material accumulates primarily in sun-exposed areas of the face, ears, neck, dorsal hands, and forearms, forming yellow-brown, dome-shaped, translucent papules and plaques [5]. These papules often become confluent, leading to a coarser texture and cosmetic disfigurement, which can significantly impact patients’ quality of life, particularly in cases with facial involvement [3].

CM is classified into five major subtypes: adult CM, juvenile CM, pigmented CM, nodular colloid degeneration (paracolloid), and acral keratosis with eosinophilic dermal deposits. Each subtype varies based on age of onset, typical clinical distribution, and associated environmental risk factors. Adult CM, the most common subtype, typically affects fair-skinned males aged 30 to 60 years who have a history of outdoor occupational exposure, such as farmers or construction workers [6]. Pathogenesis appears to involve chronic ultraviolet (UV) exposure, which induces elastin fiber degeneration within the dermis, leading to colloid accumulation. Chemical exposure to petroleum products, gas oil, and phenols may further exacerbate this degeneration, suggesting a multifactorial etiology for CM [5]. Due to its rarity and clinical overlap with other dermatological conditions, CM poses diagnostic and therapeutic challenges.

This article was previously presented as a meeting abstract at the 2025 European Academy of Dermatology and Venereology Congress on September 17, 2025.

## Case presentation

A 48-year-old female farmer with Fitzpatrick skin type III presented to the dermatology clinic with a seven-year history of asymptomatic skin lesions that had worsened mainly during the summer [7]. The patient noticed the gradual development of yellow-brown lesions on her face, neck, and dorsal hands. She reported no family history of similar lesions and had no history of drug-induced photosensitive reactions. She had been working outdoors without adequate sunscreen or sun-protective clothing for many years. She had never been exposed to petroleum products, phenols, or other chemicals.

Physical examination revealed numerous 0.1 cm to 0.3 cm, dome-shaped, nontender, yellow-brown papules confluent into plaques on the face, neck, and dorsal hands (Figures 1, 2).

**Figure 1:**
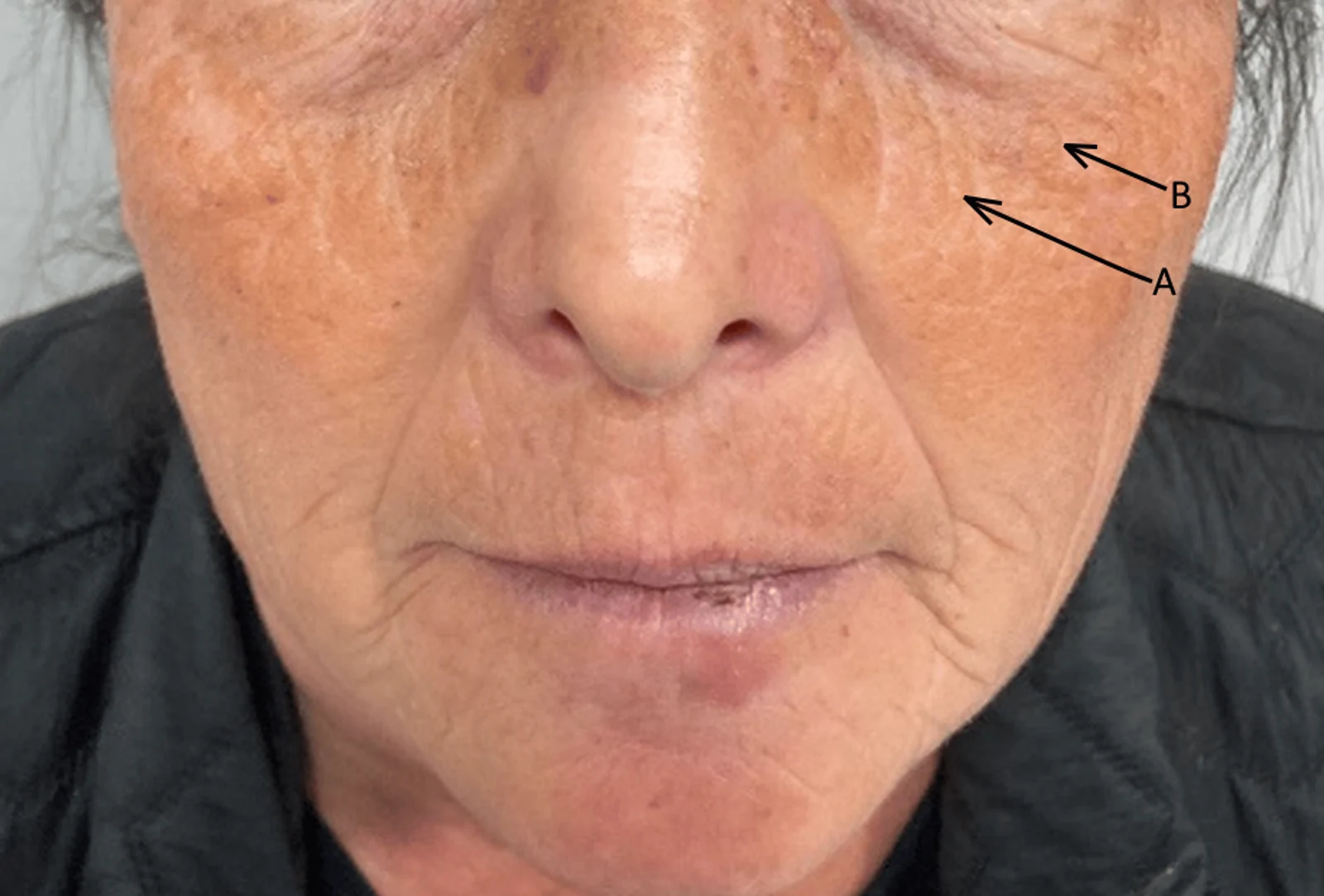
Bilateral cheeks and forehead with numerous yellow-brown papules (A) confluent into plaques (B)

**Figure 2:**
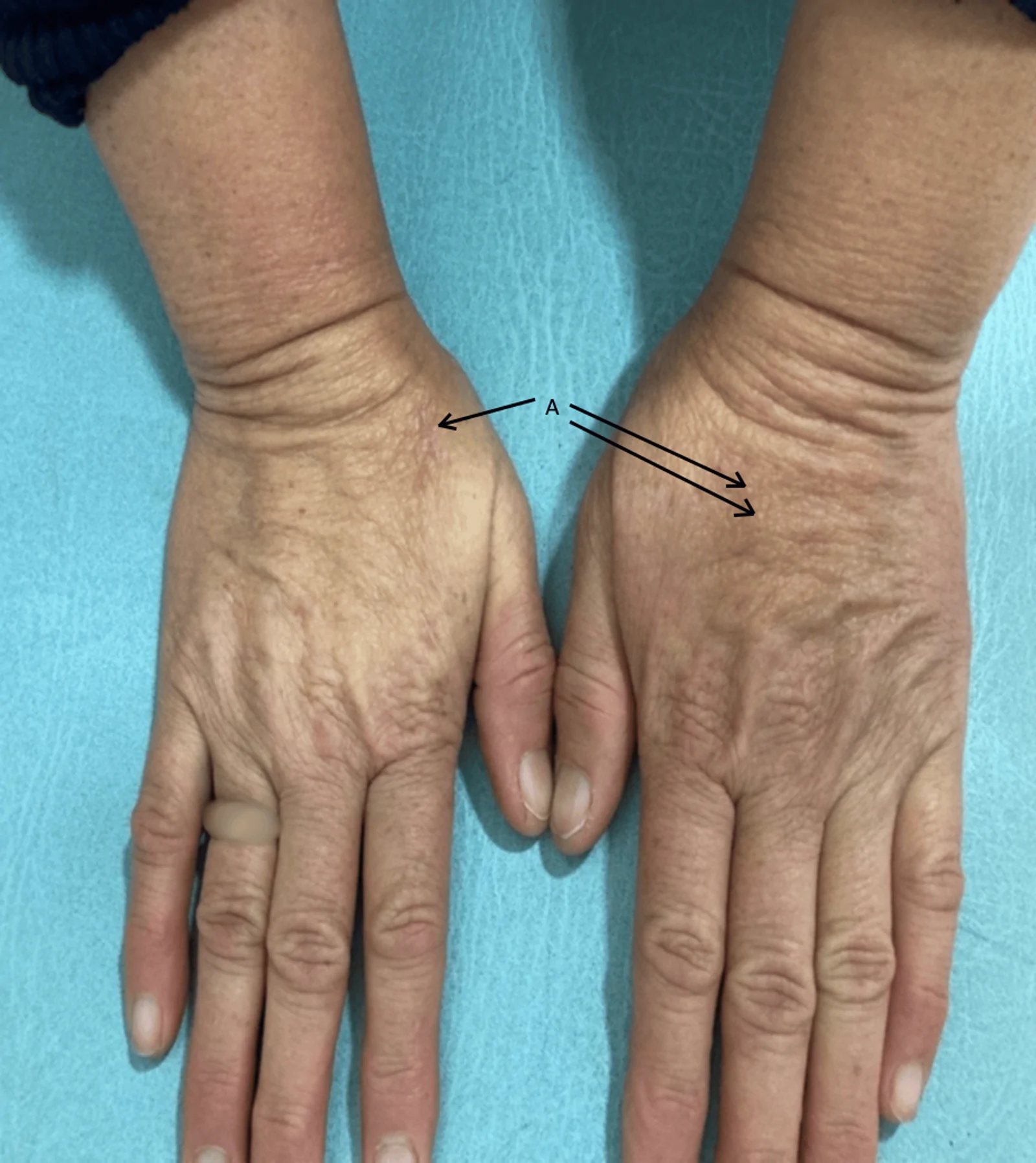
Dorsum of the patient’s hands showing numerous yellow-brown papules (A) associated with CM CM, colloid milium

The lesions displayed semi-translucency, giving a gelatinous appearance upon closer inspection. A few papules had coalesced, forming larger plaques with a roughened texture. There was no evidence of ulceration, bleeding, or secondary infection. Compression of the papules expressed a soft, golden-colored material. The patient reported mild itching when exposed to heat (Figure 3).

**Figure 3:**
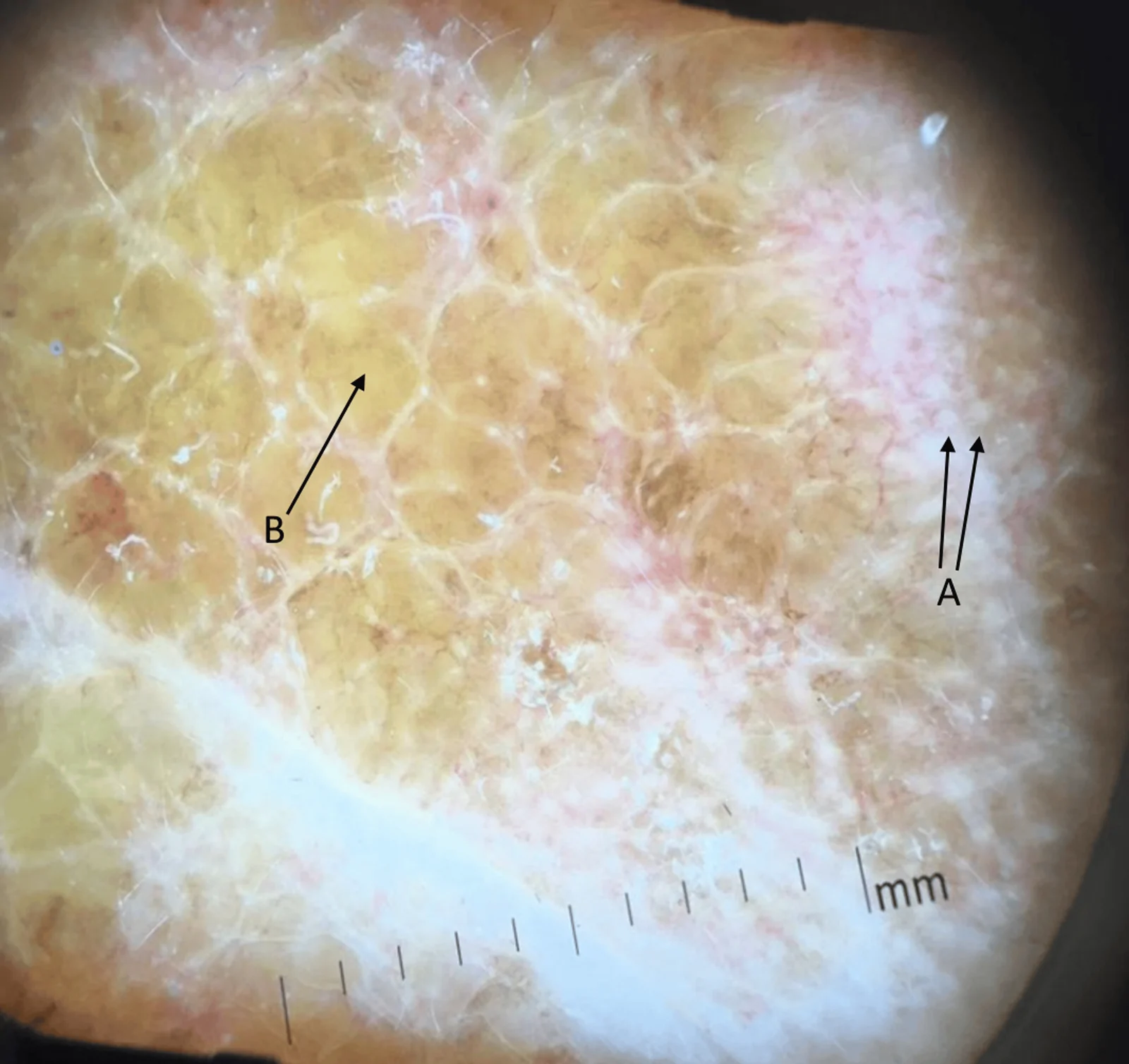
Polarized dermoscopic assessment (original magnification ×10) revealed milia-like cysts (A) on a yellowish-brown background (B)

Histopathology using hematoxylin and eosin staining of a biopsy specimen from the dorsum of the left hand revealed pale, eosinophilic, homogeneous deposits with artificial clefts in the papillary dermis, findings compatible with a diagnosis of CM (Figure 4).

**Figure 4:**
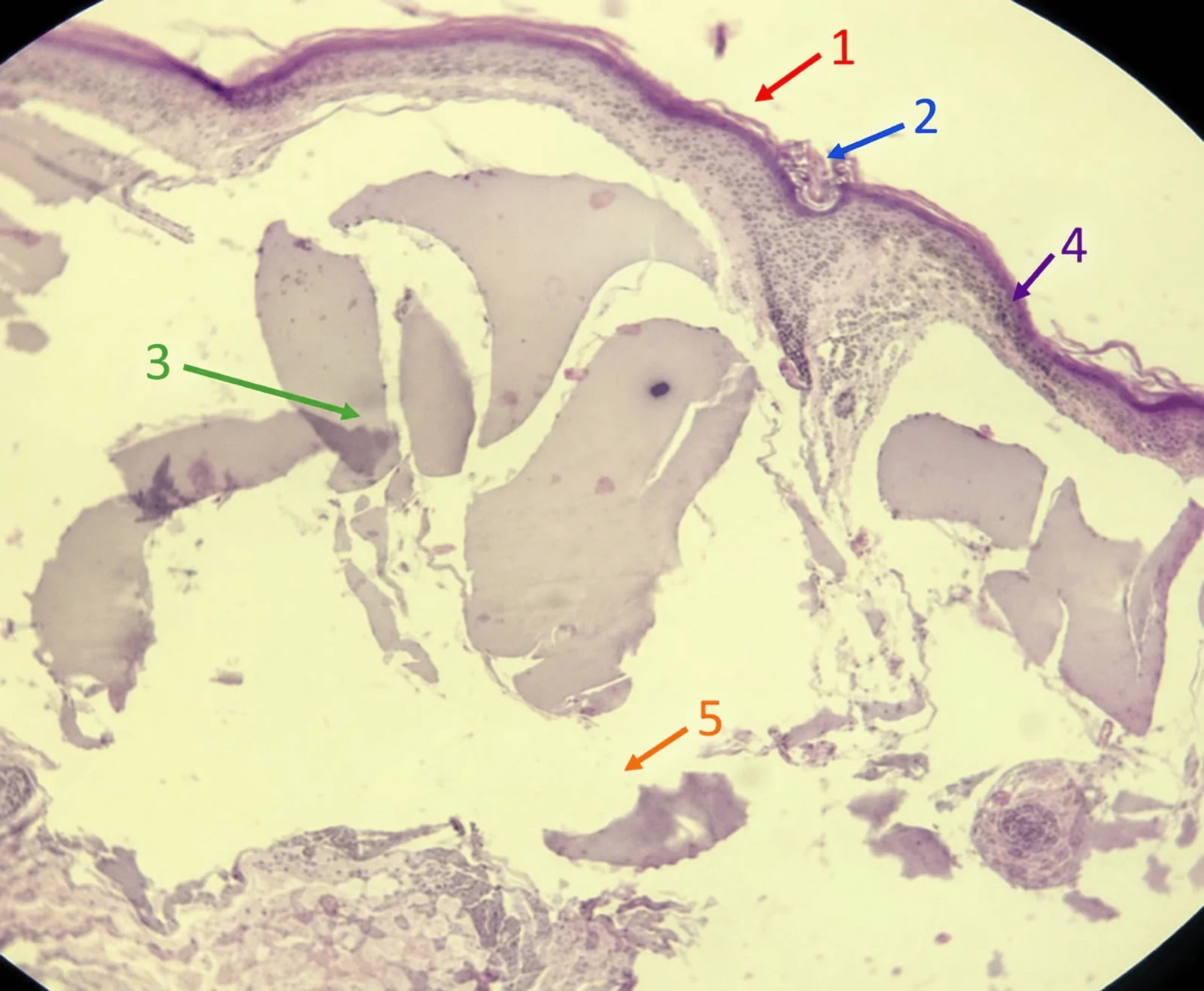
Histopathological findings of CM (1) Epidermis overlying the lesion showing mild hyperkeratosis; (2) plugging of the stratum corneum; (3) subepidermal accumulation of homogeneous eosinophilic deposits; (4) epidermal layer; (5) cleft (retraction artifact) between the colloid deposits and surrounding tissue produced during tissue processing (hematoxylin and eosin, ×100). CM, colloid milium

Initially misdiagnosed and ineffectively treated as chloasma, the condition remained unrecognized for several years, further complicating her cosmetic concerns.

Given the patient's concerns regarding the appearance of her lesions, a treatment regimen was initiated that included topical tretinoin 0.025% cream to be applied to the affected areas of the face at bedtime and urea cream twice daily for six months. Physical treatment options, such as curettage, electrosurgery, cryotherapy, photodynamic therapy, dermabrasion, CO2 laser resurfacing, and erbium laser resurfacing, were discussed, but the patient opted for medical therapy due to concerns about scarring. After three months, she reported a mild reduction in lesion thickness and a slight improvement in cosmetic appearance. Long-term follow-up was planned to assess treatment efficacy and lesion progression, with the possibility of introducing additional physical therapies if necessary. The patient was subsequently lost to follow-up; therefore, a reliable clinical assessment of the treatment response could not be performed.

## Discussion

CM’s clinical and histopathological heterogeneity reflects the complexity of its underlying pathophysiology. The deposition of colloid material in the dermis is thought to result from a degenerative process primarily affecting elastic and collagen fibers. Although the exact mechanism remains unclear, excessive sunlight and UV radiation exposure appear to be central factors, especially in adult CM. It is established that chronic sun exposure causes elastin degradation, which may subsequently impede normal elastogenesis in dermal fibroblasts. Damage to elastin fibers leads to a buildup of proteins and fibroblast products in the dermis [5]. Cellular debris accumulates in the dermis as colloid material composed of amorphous, eosinophilic proteins [8-11].

Distinct subtypes of CM (adult CM, juvenile CM, pigmented CM, nodular colloid degeneration, and acral keratosis with eosinophilic dermal deposits) further underscore the condition’s diversity.

Adult CM is the most common form of CM, primarily affecting fair-skinned individuals aged 30 to 60 years who work outdoors in occupations such as farming, where they experience high sun exposure [6]. Adult CM typically presents as clusters of flesh-colored or yellow-brown, dome-shaped papules measuring 1 mm to 4 mm in diameter. These semi-translucent papules, often merging into larger plaques, appear on sun-exposed areas, including the face, ears, neck, hands, and forearms, and may even show unilateral involvement in outdoor workers, such as taxi and truck drivers [6]. Pressure applied to the papules can express a soft, golden, gelatinous material, and the surrounding skin is often thickened and hyperpigmented [12]. Although generally asymptomatic, these lesions can sometimes cause mild itching [13]. Although CM may become more severe and extensive over the years, most cases reach their maximum development within three years and then remain unchanged [5].

Given that juvenile CM arises without significant sun exposure, genetic factors may play a more prominent role in this subtype, perhaps contributing to keratinocyte degeneration or elastin disruption independent of UV damage. Immunohistochemical studies reveal positive staining for keratin in colloid deposits, suggesting a potential role for keratinocyte products in juvenile forms [12]. This form is histologically similar to the adult type but may resolve in adulthood.

Pigmented CM is generally associated with the prolonged use of hydroquinone-based skin creams in conjunction with high sun exposure, leading to ochronosis and the development of grayish-black papules [13]. Pigmented CM has also been reported in the absence of hydroquinone exposure. In 2015, Akhyani et al. reported a case of CM in a patient who had a long history of exposure to sun and fertilizers, with no history of using hydroquinone bleaching creams [14].

Nodular colloid degeneration is a rare type, typically presenting as single nodules up to 5 cm in size, usually on the trunk or scalp [12]. Acral keratosis with eosinophilic dermal deposits, a rare form found primarily in elderly individuals, presents as painless, slowly progressive, hyperkeratotic papules on the palmar or dorsal aspects of the fingers and exhibits unique histological characteristics without the classic elastotic degeneration [15].

Diagnosis of CM relies on a combination of light microscopy, immunohistochemistry, and electron microscopy examination of a biopsy sample. Histological examination reveals fissured eosinophilic colloid masses within the papillary dermis, accompanied by solar elastosis and elastin fibers separating the lesion from the epidermis, although these may be absent in the juvenile form of the disease [1]. Amyloid stains are negative or equivocal. Immunohistochemistry with anti-keratin antibodies shows positive staining, particularly in juvenile CM, supporting keratinocyte involvement in its pathogenesis. Electron microscopy reveals granular, electron-dense material with remnants of desmosomes and binding proteins [16,17]. This combination of microscopic and immunohistochemical findings is essential for an accurate diagnosis, differentiating CM from other dermal deposition diseases, including cutaneous amyloidosis, syringomas, and actinic reticulosis.

Treatment options for CM are largely unsatisfactory, as no consensus has been reached regarding optimal management. Physical therapies, such as cryotherapy, laser resurfacing, and dermabrasion, have been employed with mixed outcomes, often leading to recurrence or poor cosmetic results. Some authors report promising results with photodynamic therapy using methyl aminolevulinate as a photosensitizer, which may reduce lesion recurrence by facilitating deeper tissue penetration [18]. Topical agents such as retinoic acid have been explored but are generally limited by their superficial efficacy and potential for irritation, particularly in patients with extensive sun-damaged skin.

The prognosis for CM varies by subtype; adult CM tends to stabilize after reaching maximal development, typically within three years, whereas juvenile forms may resolve spontaneously as patients age [5,12].

The presented case highlights the ongoing challenge in managing CM, particularly for patients exposed to occupational risk factors, such as sunlight and petroleum products. This case also underscores the need for further research into the cellular and molecular mechanisms driving colloid deposition and degeneration, as well as exploration of novel treatment modalities. Photodynamic therapy and laser technologies could provide future avenues for more effective and cosmetically favorable outcomes. Additionally, understanding the potential genetic components in juvenile CM may offer insights for early detection and management strategies in familial cases. Ultimately, CM exemplifies the complex interplay of environmental, genetic, and occupational factors in dermatologic diseases, calling for a tailored, patient-centered approach to management and care.

## Conclusions

CM is a rare dermatological disorder that requires meticulous clinical and histological evaluation for an accurate diagnosis, especially when mimicking other cutaneous disorders such as amyloidosis, syringomas, chloasma, and actinic reticulosis, making diagnostic differentiation particularly challenging. The case highlights the importance of comprehensive histopathological assessment in rare dermatological disorders and the need for individualized management strategies based on both diagnostic certainty and patient expectations.
